# Reservoirs of spoilage fungi in the built environment of cultured dairy facilities vary by season and location

**DOI:** 10.1128/spectrum.00413-26

**Published:** 2026-07-17

**Authors:** Shuai Man, Sarah E. Daly, Xiaoxuan Shi, Jingzhang Feng, Abigail B. Snyder

**Affiliations:** 1 Department of Food Science, Cornell University 171522, Ithaca, New York, USA; Connecticut Agricultural Experiment Station, New Haven, Connecticut, USA

**Keywords:** dairy processing environments, cultured dairy products, spoilage, environmental monitoring, yeasts and molds, fungal ecology

## Abstract

The processing environment is a source of spoilage fungi in cultured dairy products. However, fungi within the built environment (i.e*.,* non-food contact and architectural structures) of dairy processing facilities have not been well characterized. This study identified the seasonal and spatial variations in fungal identity, relative abundance, and diversity within the built environment of three cultured dairy facilities. Furthermore, we compared fungal genera in spoiled cultured dairy products to fungal genera in their facility’s built environment. We used internal transcribed spacer sequencing to characterize fungi from 119 environmental surfaces in three cultured dairy facilities and from 154 spoiled cultured dairy products manufactured in those facilities. The fungal genera *Aureobasidium*, *Candida, Cladosporium, Mucor, Penicillium*, and *Yarrowia* were identified in both spoiled products and their corresponding built environment. The fungal diversity on environmental surfaces varied (0–3.98, Shannon diversity; 0–176, Chao1 diversity) and was significantly (*P* < 0.01) different among Production Area (Warehouse, Raw, Filling, and Post-Pasteurization) and Season. Furthermore, the abundance of several fungal genera related to dairy product spoilage was significantly different (*P* < 0.05) among Production Area, Season, Object Type, and Facility. Network analysis further demonstrated distinct fungal composition among Production Areas within each facility. For Object Types, the relative abundance of spoilage fungal genera varied from 0% to 100%, and yeast and mold (YM) counts ranged from 0 to 8.27 log_10_ CFU/sponges. Swab sites with elevated relative abundances of spoilage genera and YM counts were identified as fungal reservoirs, which could serve as point sources for product contamination.

**IMPORTANCE:**

Spoilage of cultured dairy products from fungal contamination results in food waste and economic loss. Fungi from the facility’s built environment can be transferred to the product via air currents, water droplets, or through surface contact. Our year-long study identified overlap among fungal genera on environmental surfaces and spoiled products in three cultured dairy facilities, suggesting an overlap between environmental presence and product contamination. Furthermore, fungi was spatially differentiated within each facility, with some specific sites serving as potential point sources for product contamination. These results support the need for hygienic zoning in food processing facilities. Our work confirms the importance of environmental surface swabbing as a tool to locate potential fungal reservoirs in the built environment, which can aid targeted interventions against spoilage fungi in dairy processing facilities.

## INTRODUCTION

Spoilage in dairy products can result in economic loss and food waste (1, 2). Cultured dairy products can be contaminated with yeasts and molds post-pasteurization, resulting in spoilage during shelf life (3). Since many fungi tolerate low pH and temperature, they can survive and proliferate in pasteurized cultured dairy products during refrigerated storage (4–6). Growth of spoilage fungi can decrease the quality (i.e*.,* odor, taste, and appearance) of dairy products. Several fungal genera, including *Alternaria, Aureobasidium, Candida*, *Cladosporium, Exophiala, Geotrichum, Mucor*, and *Penicillium*, are prevalent in dairy processing environments and have been linked to product contamination and spoilage (5, 7–10).

There are multiple routes through which fungi in the processing environment can contaminate products. Fungi on non-food contact surfaces, such as floors, walls, doors, and ceilings, can contaminate exposed products via water droplets (i.e*.*, condensation and leaks), water sprays used for cleaning, surface-to-surface contact, or activities of personnel (11, 12). Moist, humid corners and crevices of food processing facilities are especially susceptible to fungal growth (13–15). Filamentous fungi (i.e*.*, molds) on surfaces can contaminate products via spores generated during their lifecycle, which are diffused into air currents by fans and ventilation units and disseminated into exposed product (16–19). Diffusion via air is typically proposed as the predominant route for mold contamination of the product. Fungi in the air can also settle on surfaces (20) and thereby influence fungal presence within the processing environment. For example, studies have found the same species of *Penicillium* and *Cladosporium* within the dairy processing environment’s surfaces, air, and product (18, 21). Given these multiple routes of contamination, even surfaces that are not in close proximity to spoiled product should be investigated as potential sources of product contamination.

In food manufacturing, “architectural structures,” such as floors, walls, ceilings, doors, and ventilation units (i.e*.*, the built environment), can harbor yeasts and molds since they are exposed to varying humidity and surface moisture levels and receive minimal sanitation (22–24). However, these structures are understudied as sources of fungal contamination in food manufacturing, with food processing equipment often being prioritized in sanitation regimes to reduce product contamination (25). Given the multiple routes by which fungi can be dispersed in the processing environment, even structures distant from food preparation areas could be a potential source of spoilage fungi in products. Therefore, identifying fungal reservoirs (i.e*.*, locations that harbor spoilage fungi) in the built environment can help inform sanitation strategies to reduce product contamination.

This research identifies and examines the fungal ecology in the built environment of three cultured dairy facilities. We used internal transcribed spacer (ITS) sequencing and traditional culturing methods to (i) characterize and compare the identity of fungal genera within the built environment of cultured dairy facilities to spoiled products manufactured in those facilities, (ii) investigate fungal identity, relative abundance, diversity, and distribution (i.e*.*, fungal ecology) within the built environment across seasons, facilities, production areas, and objects, and (iii) identify potential reservoirs of spoilage fungi within the built environment. Our study provides a nuanced understanding of how fungal ecology is affected by seasonal and spatial variations in dairy processing environments, which can inform targeted and effective spoilage prevention strategies.

## RESULTS AND DISCUSSION

### Yeast and mold genera in spoiled products were identified in the built environment

**Fig 1:**
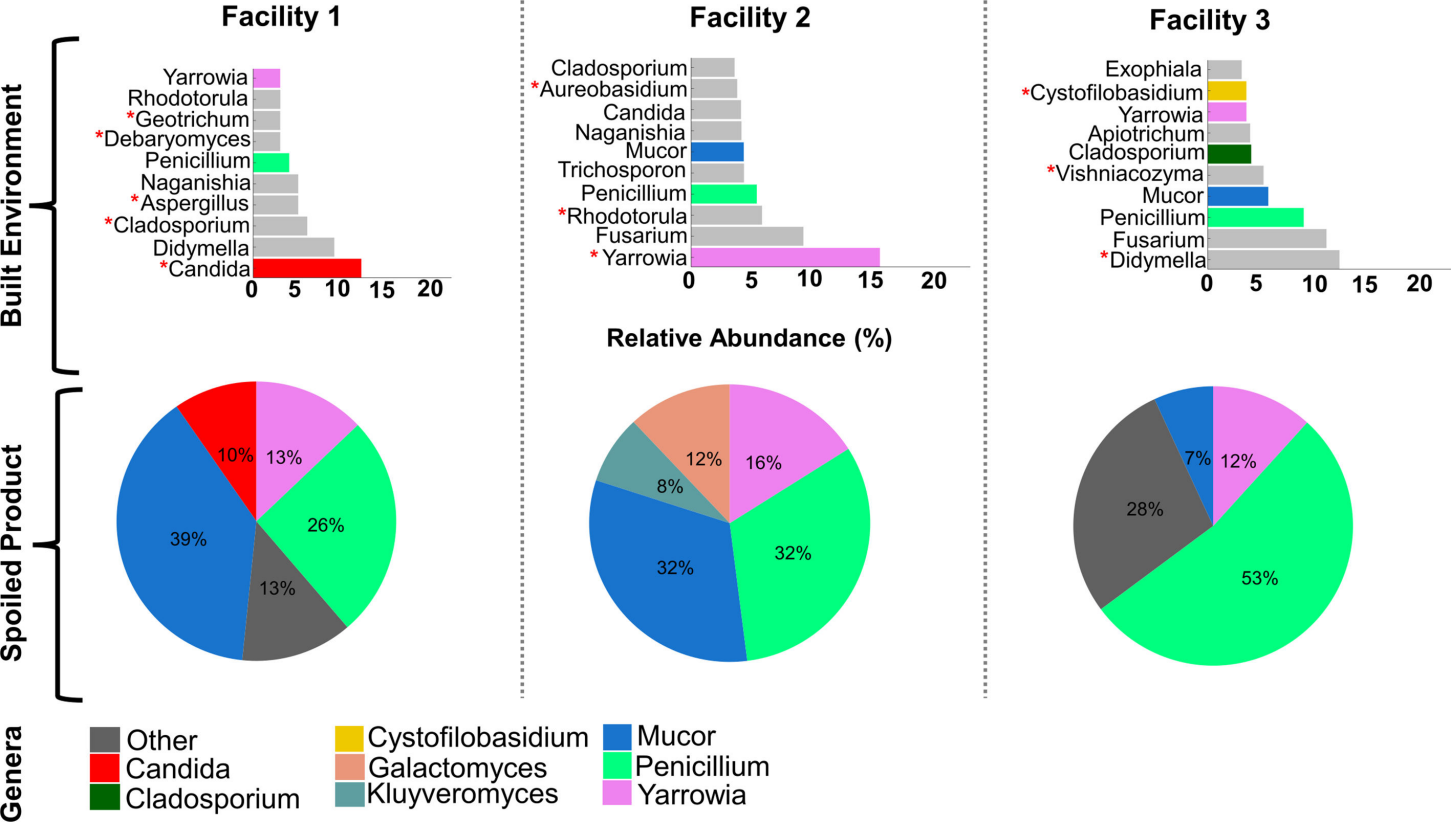
Built Environment: the most relatively abundant fungal genera within the built environments of Facilities 1, 2, and 3. Colored bars indicate that the genera were also in the spoiled product from that facility. * indicates differentially abundant fungal genera. Spoiled Product: percentage of fungal genera among all isolates from all spoiled products manufactured in each facility. The sectors for “Other” in pie charts were genera that were present in <5% of isolates (*n* = 201).

Among the environmental surfaces of all three facilities, 32 fungal genera above 1% relative abundance were identified (Table S1). Several spoilage genera were abundant across every facility’s built environment, including *Cladosporium, Fusarium,* and *Penicillium* (Fig. 1). However, each facility’s built environment had several distinct differences in the identity and relative abundance of top fungal genera.

The genera *Aspergillus, Candida, Debaryomyces,* and *Rhizopus* were significantly more abundant on Facility 1 surfaces (Fig. 1). In Facility 2, *Sarocladium, Naganishia*, and *Yarrowia* were significantly more abundant on surfaces (Fig. 1). *Cutaneotrichosporon* and *Exophiala* were significantly more abundant on Facility 3 surfaces (Fig. 1).

Facility 3 had the highest average yeast and mold (YM) counts of all three facilities (3.64 ± 2.26 log_10_ CFU/sponge) (Table S2). Furthermore, YM values ≥5 log_10_ CFU/sponge were overrepresented in Facility 3 (Fig. S1). Most likely, this was because Facility 3 was consistently associated with high humidity (>60%) and detectable surface moisture (Fig. S1). Indoor humidity can increase surface moisture, which is conducive to the growth of YM (17, 26). Older buildings, like Facility 3, have been positively correlated with fungal presence on interior surfaces and are more likely to have higher indoor humidity and moisture due to older air handling and ventilation systems (27–30).

Across the 166 received products, 154 had fungal isolates and were considered to be “spoiled.” Among all spoiled products, there were 201 fungal isolates and 24 unique genera (Table S3). Many fungal genera commonly associated with cultured dairy product spoilage were frequently identified in spoiled products, including *Candida, Mucor, Penicillium,* and *Yarrowia* (Fig. 1). Other spoilage genera, which comprised under 5% of isolates, included *Aureobasidium* and *Rhodotorula* (Table S4). Facility 3 had the most unique genera isolated in spoiled products (*n* = 20), probably because Facility 3 provided the most products (*n* = 129) (Table S4).

Several fungal genera isolated from spoiled products were identified in the corresponding facility’s built environment. Across all facilities, *Penicillium* and *Mucor* were the most frequently isolated genera from all products that were also commonly present in the built environment (Fig. 1). In Facility 1, *Mucor* comprised 38.7% of product isolates and had an average relative abundance of 3.2% on surfaces, while *Penicillium* comprised 25.8% of product isolates and had an average relative abundance of 7.4% on surfaces (Fig. 1; Table S4). In Facility 2, *Penicillium* comprised 32.0% of product isolates and had an average relative abundance of 5.4% on surfaces, while *Mucor* comprised 32.0% of product isolates and had an average relative abundance of 2.0% in the built environment (Fig. 1; Table S4). In Facility 3, *Penicillium* comprised 53.1% of product isolates and had an average relative abundance of 7.2% on surfaces, and *Mucor* comprised 6.9% of isolates and had an average relative abundance of 2.3% on surfaces (Fig. 1; Table S4).

Several other genera identified in spoiled products were also identified in their facility’s built environment. *Yarrowia* comprised 16.0% of Facility 2’s product isolates and had an average relative abundance of 20.6% on Facility 2’s surfaces (Fig. 1; Table S4). In Facility 1 products and surfaces, *Yarrowia* comprised 12.9% of product isolates and had an average relative abundance of 3.0% on surfaces (Fig. 1; Table S4). *Candida* had an average relative abundance of 11.0% on surfaces in Facility 1 and comprised 9.7% of isolates from spoiled products from Facility 1 (Fig. 1; Table S4). The genera *Aureobasidium*, *Cladosporium*, and *Cystofilobasidium* were also in both the built environment and spoiled products in Facility 3 (Table S4).

Not all of the fungal genera identified in spoiled products were found in their facility’s built environment. For example, *Yarrowia* had less than 1% average relative abundance in the built environment of Facility 3 though it comprised 11.7% of spoiled product isolates (Fig. 1; Table S4). In addition, *Kluyveromyces* comprised 8.0% of isolates in Facility 2, but the relative abundance, on average, was below 1% on Facility 2 surfaces (Fig. 1; Table S4). By contrast, there were several fungal genera that were abundant on surfaces but not in spoiled products. Both *Aspergillus* and *Didymella* were abundant on surfaces in all three facilities but were not identified in spoiled product (Fig. 1). *Cladosporium* was within the top 10 most abundant genera in the built environment of all three facilities but was only isolated from spoiled products from Facility 3 (Fig. 1; Table S4).

There are several possible explanations for these discrepancies. It is possible that these fungi, which were on architectural structures at some distance from food contact surfaces, were less likely to contaminate the product. Fungi from other sources not examined in this study could have contaminated the product, including food processing equipment surfaces, product ingredients, starter cultures, or via the air during packaging (31–33). The genus *Penicillium* is prevalent in indoor air and is one of the most common genera isolated from production environments and spoiled dairy products (5, 9, 18). Additionally, some species of fungal genera, including species of *Candida* and *Yarrowia*, are common in dairy products and do not threaten product quality (16, 34). Furthermore, fungi can be introduced post-pasteurization through the addition of product ingredients such as fruit preparations (35). Notably, several of the spoiled products sampled in this study were fruit-flavored yogurts (Table S5), which could have been contaminated through fruit additions after pasteurization. Alternatively, fungi from the built environment may have contaminated the product but were not competitive in the yogurt matrix, which often includes preservatives or protective cultures. Future studies should work to pinpoint the exact stage of processing where the spoilage fungi contaminate the product.

The data generated in this study are insufficient to establish precise routes of cross-contamination between the product and the built environment. The relative abundance values from the built environment were derived from culture-independent amplicon sequencing, while isolates were identified after culturing. This is an important distinction, as culture-independent sequencing can detect both viable and non-viable microbial cells (36, 37). Therefore, fungi identified on surfaces may not have necessarily been able to cause product spoilage. Despite these limitations, the results suggest an overlap between genera isolated from spoiled products and genera abundant on environmental surfaces. The significance of the observed overlap suggests that the analysis of spoiled product has its value as a contextualization tool of so-called “spoilage genera.” This suggests that the built environment may be an important source of spoilage fungi in dairy processing environments.

### Fungal ecology in the built environment varied by Season and Production Area

**Fig 2:**
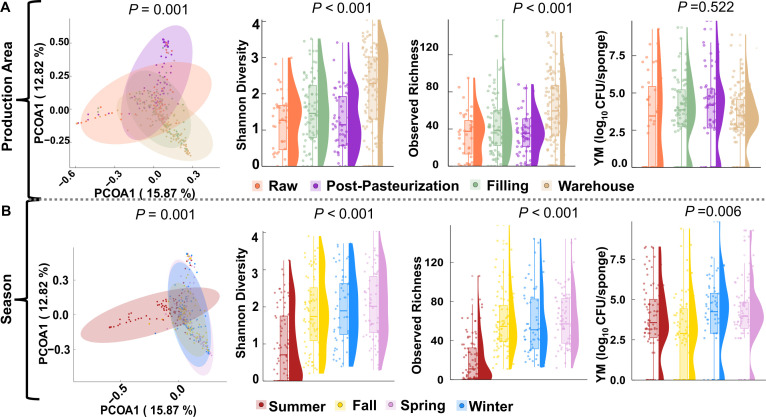
Relative abundance of fungal genera identified on built environment surfaces across (**A**) Production Areas and (**B**) Seasons. Only genera above 5% relative abundance were included. * indicates a significant difference in the abundance of that genus.

**Fig 3:**
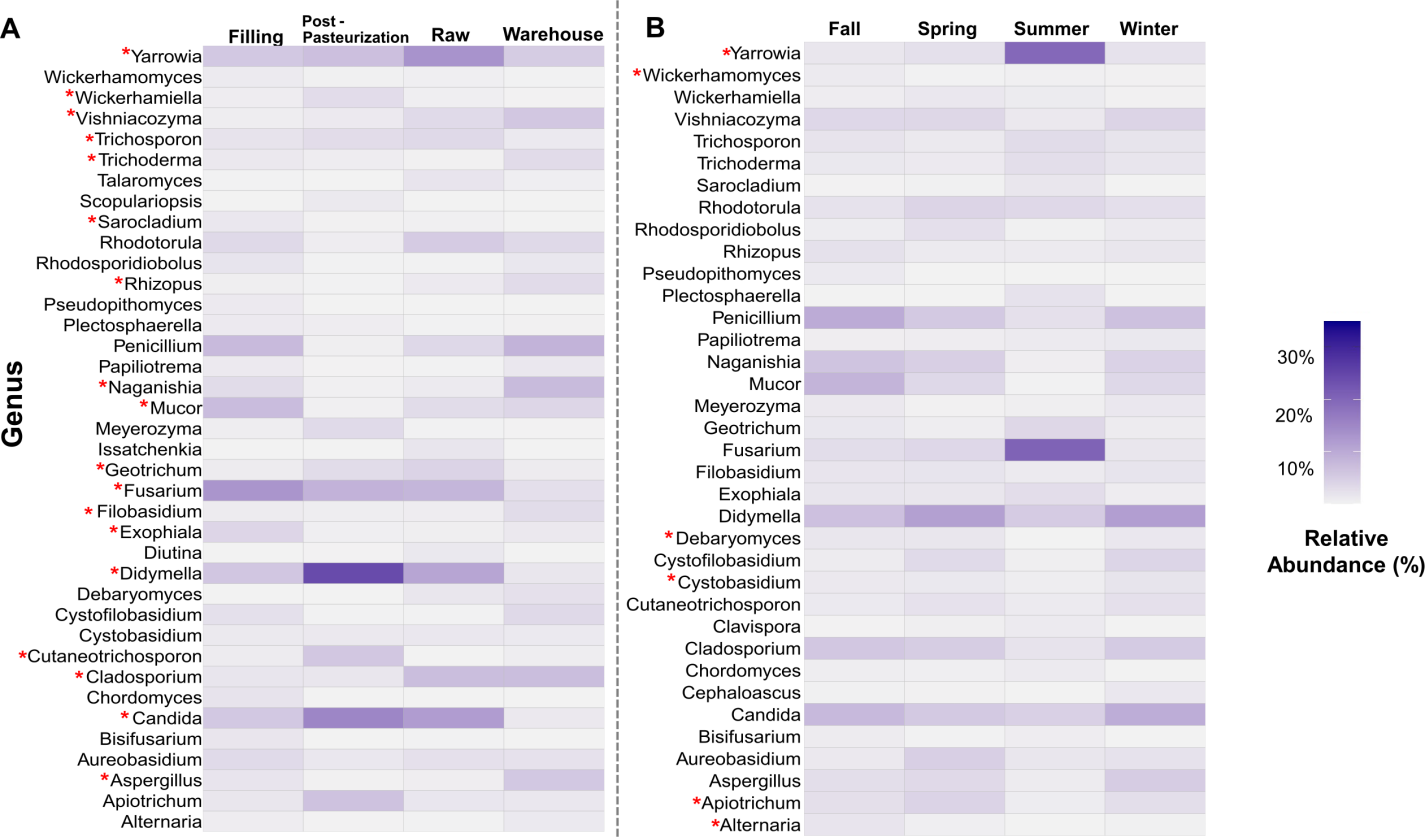
Principal coordinate analysis plots of fungal beta diversity within the built environment, colored by (**A**) Production Area and (**B**) Season. Boxplot and violin plot of Shannon diversity and YM count, with the width of the violin indicating the distribution of individual measurements by (**A**) Production Area and (**B**) Season. The colors represent Production Area or Season.

The fungal diversity (alpha and beta) in the built environment of cultured dairy facilities varied significantly by Season (Fig. 3B). Surface fungi collected during the Summer season across all facilities formed a distinct cluster compared to the other seasons (Fig. 3B). Additionally, fungi collected from surfaces in the Summer had the lowest average Shannon diversity (0.88 ± 0.98) (Table S2). Notably, *Aspergillus, Cladosporium, Mucor*, and *Penicillium* had the lowest relative abundances in the Summer compared to the other seasons (Fig. 2B). In fact, *Aspergillus* was significantly more abundant in the Winter (Fig. 2B). Meanwhile, the genera *Fusarium* and *Yarrowia* had the highest relative abundances in the Summer (Fig. 2B). There were numerical but not statistical differences in YM counts by Season, with Spring and Summer exhibiting higher average YM counts (3.70 ± 1.78 and 3.41 ± 2.17 log_10_ CFU/sponge, respectively) compared to Fall and Winter (2.56 ± 2.10 and 2.95 ± 2.37 log_10_ CFU/sponge, respectively) (Table S2). The high temperature and humidity levels during summer months can promote fungal growth and sporulation (38–41), which can result in increased spoilage incidents (15). Furthermore, seasonal conditions can encourage or inhibit the growth of specific fungal genera. For example, *Aspergillus* and *Penicillium* have been shown to peak in indoor air during winter times (42), explaining their lower relative abundances in the summer in our study. Our results indicate that quantifying seasonal fluctuations in fungal loads can provide insight into fungal presence in dairy processing environments.

The relative abundance of spoilage genera across all facilities was significantly different among Production Areas. The genera *Mucor, Exophiala*, *Fusarium,* and *Sarocladium* were significantly abundant on surfaces in Filling areas (Fig. 2A). *Cutaneotrichosporon* was significantly abundant on Post-Pasteurization surfaces, and *Cladosporium* was significantly more abundant on Warehouse surfaces (Fig. 2A). The genera *Aureobasidium*, *Geotrichum,* and *Yarrowia* had the highest relative abundances on Raw area surfaces (Fig. 2A; Table S7). The “machinery mold,” *Geotrichum,* has been isolated from surfaces in food processing environments and is a source of concern for post-processing contamination (43). Other fungal genera of particular concern were *Aureobasidium* and *Exophiala*, also known as “black yeasts,” which can exhibit high stress tolerance and the ability to adhere to surfaces through extracellular polysaccharide secretion (44). These attributes can enable *Aureobasidium* and *Exophiala* to thrive in food production environments (45, 46). Furthermore, the relative abundance of *Aureobasidium* was significantly higher in Spring (Fig. 2B), indicating that favorable seasonal conditions can further increase *Aureobasidium* presence. Further investigation of the influence of season and location on specific fungal genera growth in dairy processing environments is warranted.

Fungal diversity (alpha and beta) of the built environment across all facilities was significantly different among Production Areas (Fig. 3A). Warehouse areas had the highest average alpha diversity values (Shannon = 2.01 ± 1.01 and Chao1 = 71 ± 49) (Table S2). However, Filling and Post-Pasteurization areas had the highest average YM counts (3.52 ± 1.69 and 3.64 ± 2.66 log_10_ CFU/sponge, respectively) (Table S2). The Post-Pasteurization areas, where product was transported and sealed into packaging, also had the second highest average Chao1 richness (47 ± 32) and Shannon diversity (1.28 ± 0.95) (Table S2). Transport equipment and packaging materials moving through the Post-Pasteurization and Warehouse areas could have introduced different fungal genera into these environments, thereby increasing microbial diversity (24, 47). Furthermore, cleaning was infrequent in Warehouse areas and was completed mainly through sweeping (Table S3). With infrequent cleaning, fungi can accumulate on surfaces over time, contributing to fungal diversity. The presence of spoilage genera in Filling and Post-Pasteurization areas increases the risk of product contamination, since these areas had uncovered cheese vats and yogurt filling lines (Table S8).

**Fig 4:**
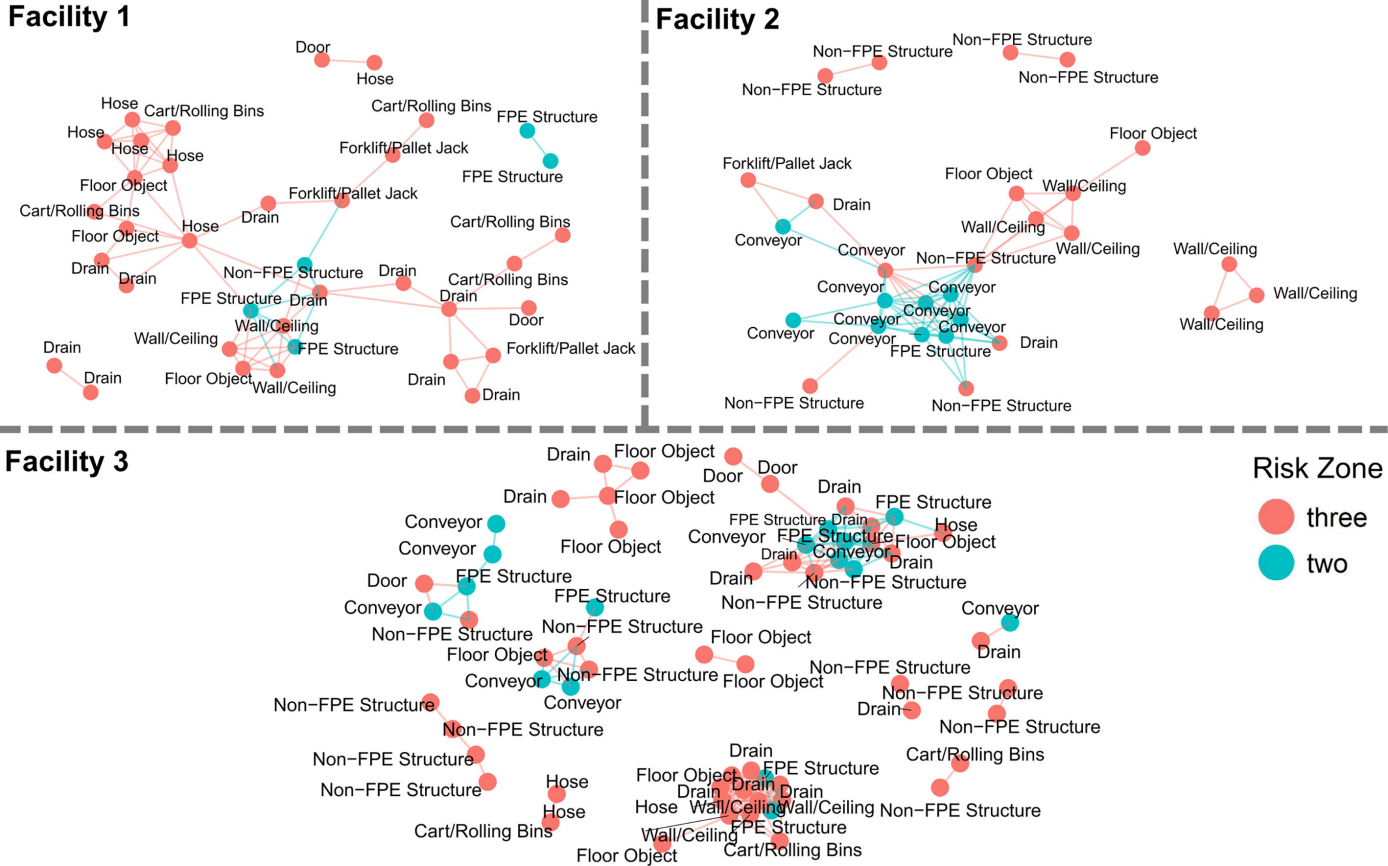
Networks of environmental surface fungi within Facilities 1, 2, and 3. Nodes (points) correspond to individual surface swabs, and edges (lines) connect swab sites based on their similarity in fungal composition calculated using the Bray-Curtis dissimilarity metric. Nodes were connected if they had >20% similarity. The colors represent the location (Production Area) where the surface swabs were collected. Clusters indicate that many of the fungal taxa within those individual surface swabs co-occurred (i.e., positively correlated).

Network analysis also revealed environmental surface fungi clustering by Production Area. Across all facilities, surface swabs from Warehouse areas were particularly interconnected (Fig. 4). Thus, most surfaces from Warehouse areas within each facility had similar fungal composition. Facility 1 was the most highly centralized network, with compact clusters and most nodes connected within a single, large central cluster. In Facility 1, each Production Area had specific environmental conditions that most likely influenced and shaped the fungal ecology on those surfaces. These results further reveal the spatial differentiation of fungi in dairy processing environments, which supports the importance of hygienic zoning in food processing facilities. Zoning in food processing facilities (48) establishes areas within food production facilities based on priority of hygiene. For example, Filling areas are considered “high-hygiene” zones because the product is most susceptible to contamination since it is exposed to the environment after the pasteurization step. Maintaining physical separation between these areas and those of lower hygienic status (e.g., Warehouse), which often have higher loads of environmental fungi, is a strategy to reduce production contamination. However, personnel, transport equipment (e.g., carts and forklifts), and materials (e.g., packaging and bulk ingredients) “cross” zones and can serve as vectors for environmental biota (48). Fungal counts have also been shown to vary substantially across production areas and objects within dairy facilities (49). The environmental surfaces in our study had variable YM counts and differed in fungal diversity, fungal identity, and relative abundance, because they also differed in sanitation practices, exposure to food products, traffic patterns, and other environmental conditions, which may have influenced the introduction and survival of fungi.

### Objects in the built environment harbored fungal reservoirs

**Fig 5:**
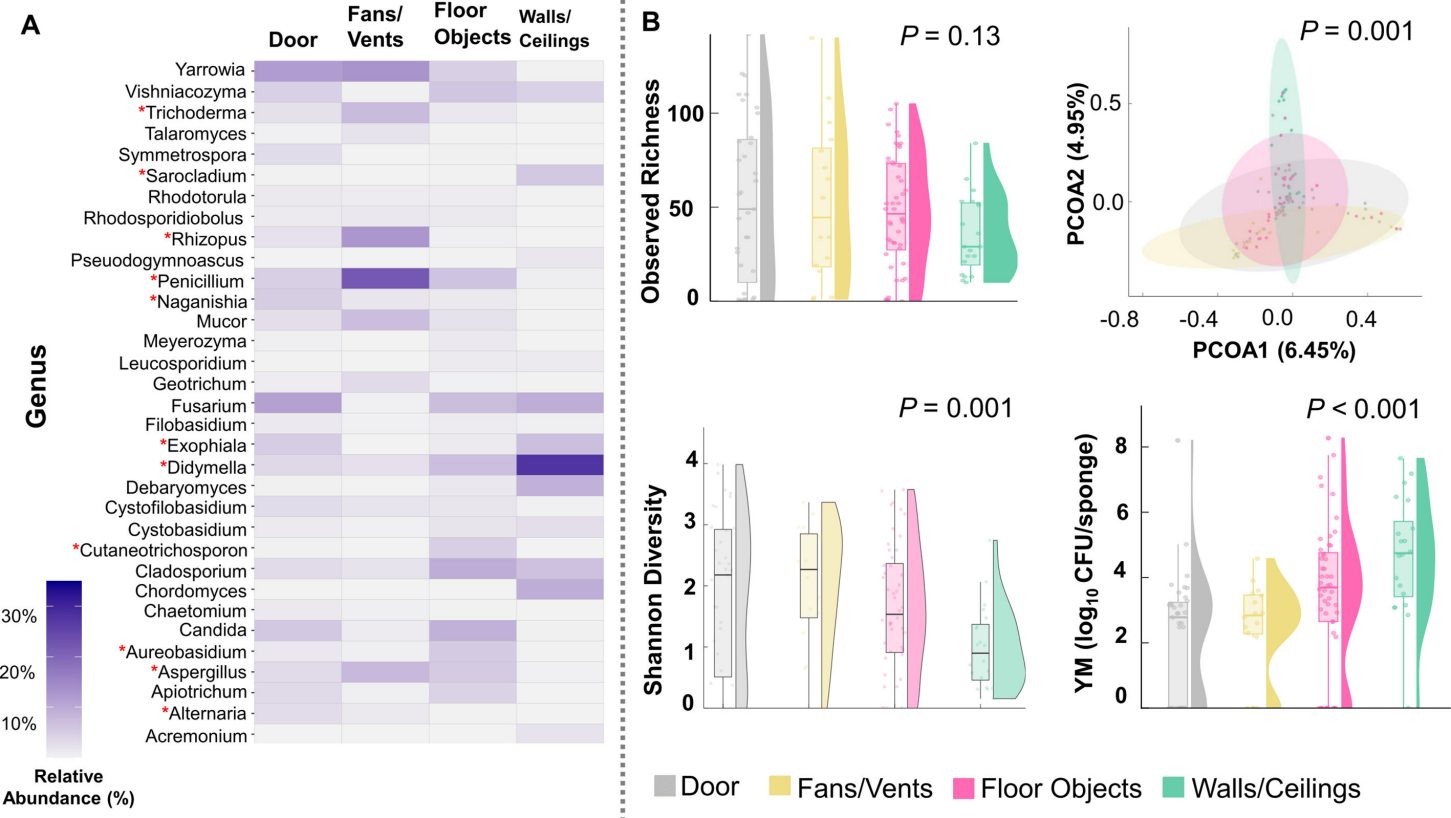
(**A**) Heatmap of relative abundance of fungal genera per Object Type. Only genera above 1% relative abundance were included. Asterisks (*) indicate significant differences in differential abundance among taxa. (**B**) A Venn diagram of the number of unique genera overlapping within surface swabs collected from each Object Type above 1% relative abundance. Principal coordinate analysis (PCoA) plots of fungal beta diversity within the built environment, colored by Object Types. Barplots: boxplot and violin plot of Shannon diversity and YM count, with the width of the violin indicating the distribution of individual measurements.

Shannon diversity, beta diversity, YM counts, and relative abundance were significantly different among Object Types across all facilities (Fig. 5B). Floor Objects and Walls/Ceilings had the lowest average Shannon alpha diversity (1.64 ± 1.06 and 1.04 ± 0.74) but the highest average YM counts (3.53 ± 2.16 and 4.66 ± 1.82 log_10_ CFU/sponge) (Fig. 5B; Table S2). Floor Objects and Walls/Ceilings were associated with wet surface conditions, which are conducive to harboring yeasts (Fig. S2). Object surfaces also contained several spoilage genera. The genera *Aureobasidium* and *Cutaneotrichosporon* were significantly more abundant on Floor Objects (Fig. 5B). The genera *Didymella, Exophiala,* and *Sarocladium* were significantly more abundant on Walls/Ceilings (Fig. 5B). Fans/Vents and Doors had lower average YM counts (2.53 ± 1.40 and 2.17 ± 1.94 log_10_ CFU/sponge) but higher average Shannon diversity values (2.03 ± 1.01 and 1.84 ± 1.32) among all Object Types (Fig. 5B; Table S2). The genus *Alternaria* was significantly more abundant on Doors (Fig. 5A). *Aspergillus, Penicillium,* and *Rhizopus* were significantly more abundant on Fans/Vents (Fig. 5A; Table S15). The Fans/Vents that we sampled were located in the Warehouse and Raw areas, so they were not in direct proximity to exposed pasteurized product. However, fans and vents have reportedly contributed to the airborne spread of molds from the built environment to product (18), as fungi in the air can also settle on surfaces (20).

The fungal ecology further varied within Object Types, with individual objects harboring reservoirs of spoilage fungi. For example, *Candida* had an average relative abundance of 11% across all Object Types in Facility 1 (Fig. 1; Table S14). The bulk of *Candida* in this facility was on Doors and Floor Objects (Fig. 5A). However, even among Doors and Floor Objects, the relative abundances among individual swab sites were skewed. For example, the relative abundance of *Candida* on a single Door swab site at Facility 1 was 100% (sample 334), while the remaining Facility 1 Door swab sites had 0%–7% relative abundance (Table S12). In addition, sample 334, located in a Post-Pasteurization area where risk of product contamination is enhanced, had a YM count of 3.16 log_10_ CFU/sponge, suggesting cell viability (Table S3). In Facility 2, three Door swab sites had over 90% (samples 288, 270, and 289) relative abundance of *Yarrowia,* while nine sites had 0% (Table S12). Only sample 270 had a non-zero YM count, suggesting that even though samples 288 and 289 had elevated relative abundances of *Yarrowia*, the cells may not have been viable. In Facility 3, two Fan/Vent swab sites (samples 140 and 141) had *Penicillium* relative abundances of 89% and 82%, while the remaining swab site (sample 142) had a 0% relative abundance. Samples 140 and 141 had 3.6 and 3.9 log_10_ CFU/sponge YM counts (Table S3), suggesting that these Fans/Vents could be disseminating viable *Penicillium* into the facility’s air. Furthermore, a single floor site (sample 79) in Facility 3 had an *Aureobasidium* relative abundance of 94% and a non-zero YM count (3.75 log_10_ CFU/sponge) (Table S3). At the remaining Facility 3 Floor sites, the relative abundances of *Aureobasidium* ranged from 0% to 14% (Table S12). Another Floor site in the Facility 3 Filling area, where product contamination risk was enhanced, had *Mucor* relative abundance of 71% (sample 50) and a non-zero YM count of 5.83 log_10_ CFU/sponge (Table S3). The remaining Facility 3 floor sites all had 0%–2% relative abundances of *Mucor* (Table S12). Hence, individual sample sites with elevated relative abundances of spoilage fungi and YM counts could serve as point sources of spoilage fungi.

**Fig 6:**
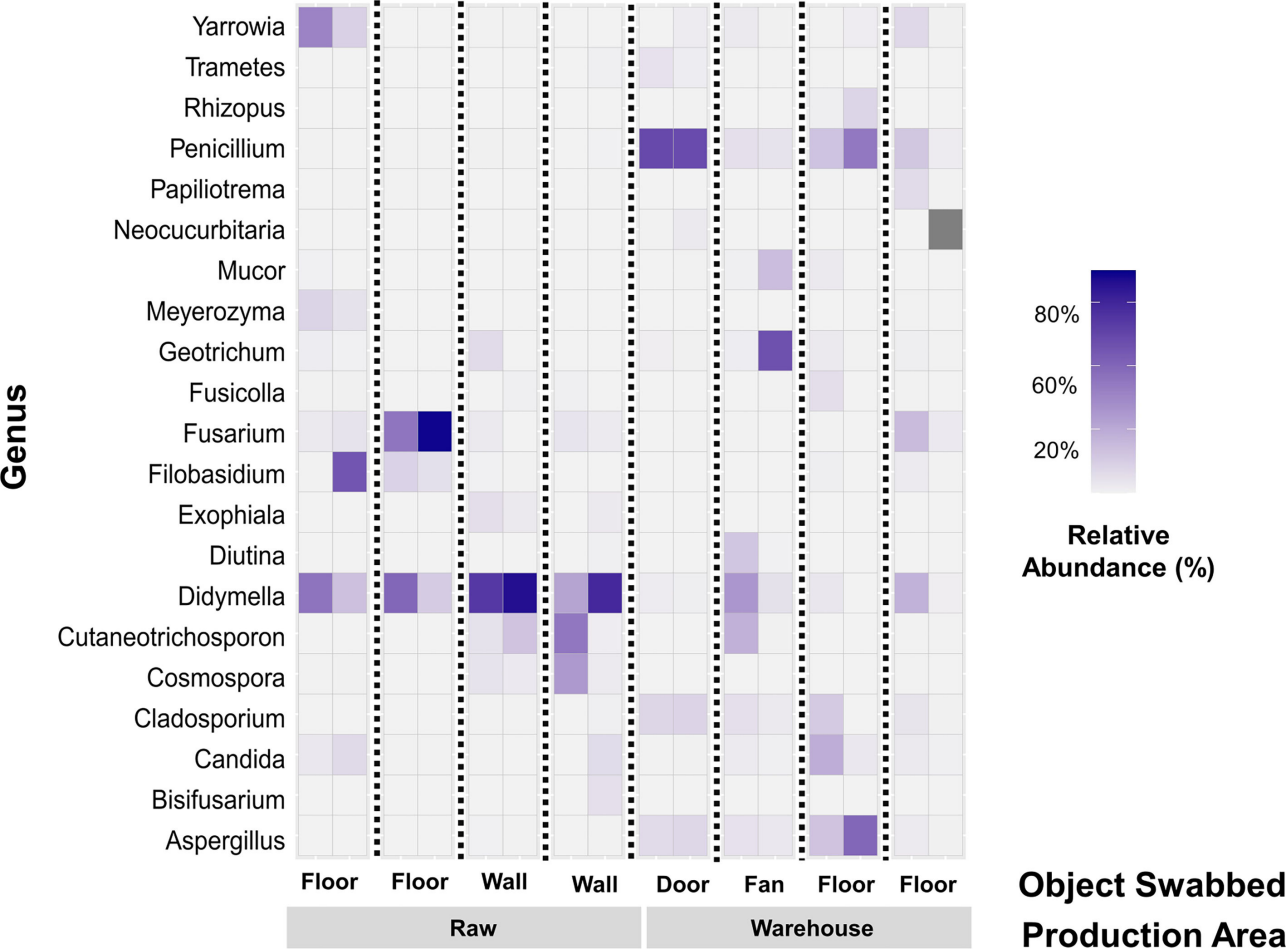
The relative abundance of fungal genera collected from side-by-side swabs of the same surface at the same sampling time point. Only fungal genera with >5% relative abundance were included.

Fungal ecology metrics further varied within different locations on the individual object. The results collected from side-by-side surface swabs at the same time illustrate the potential for variation in the identity and diversity of genera on the same object (Fig. 6). Most of the pairs had a >10% difference in diversity values and YM counts (Table S13). The relative abundance also varied between pairs (Fig. 6). For example, there were differences in *Aspergillus, Geotrichum,* and *Penicillium* relative abundances on a pair of Warehouse Fan swabs (Fig. 6). There were also differences in *Didymella, Filobasidium,* and *Yarrowia* relative abundances from paired Raw area floor swabs. Nevertheless, Door swabs had similar fungal identities and high relative abundances of *Penicillium*. These results further indicate that there is high variability among surfaces of the same object. The “Object Type” factor probably did not account enough for other surface attributes, including sub-structure, moisture, roughness, cracking, or the presence of organic matter; hence, there was still unexplained variability within Object Type.

These results are relevant to environmental swabbing programs where side-by-side swabs are collected for separate analyses but linked to the same sample site or object. For example, in research where one paired swab is used for amplicon sequencing and the other is used in an enrichment, there could be a discrepancy in the detection of a specific pathogen, such as *Listeria*. There may be limitations to the assumption that fungi present at adjacent swab sites are the same. However, pairing amplicon sequencing data with classical culturing methods and including precise metadata can thus aid in locating fungal reservoirs in the built environment. Therefore, further characterization of sub-parts and location within objects will be needed to identify and predict potential fungal reservoirs.

**Fig 7:**
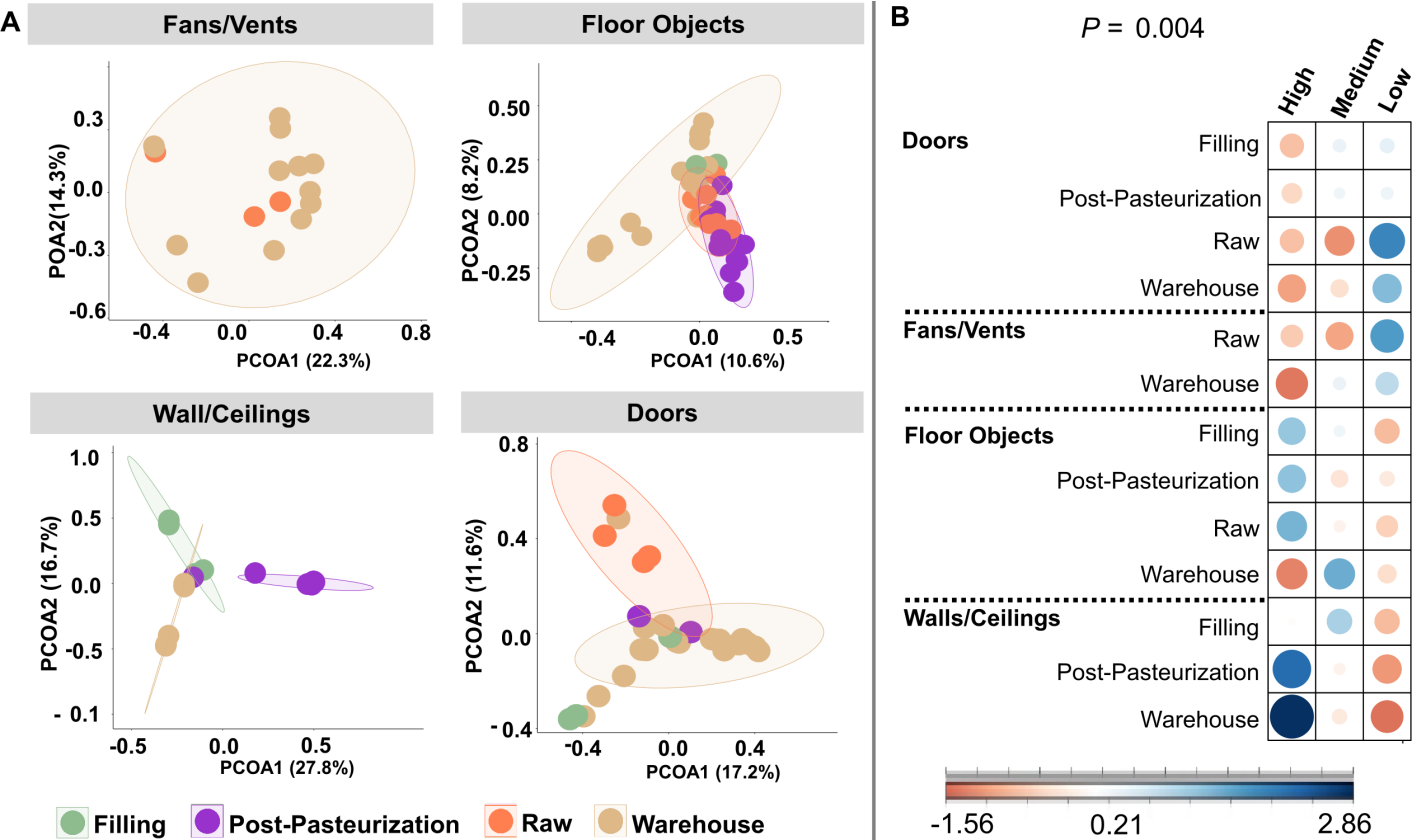
(**A**) Principal coordinate analysis (PCoA) plots of fungal beta diversity within the built environment, colored by Production Area for each Object Type. (**B**) Residuals from the Chi-squared test of Object-Production Area and YM count category (*P* = 0.004). Blue circles represent a positive association, and red circles represent a negative association. The diameter and color intensity of the circle correspond to the strength of the association. The larger and darker the circle, the stronger the relationship.

Overall, the fungal ecology of the built environment could be associated with the spatial and seasonal factors we developed. Fungi in the built environment displayed significant differentiation across Facility, Production Area, Season, and Object Type based on Bray-Curtis dissimilarity (permutational multivariate analysis of variance [PERMANOVA]) (*P*  <  0.001) (Table S9). Production Area, Object Type, Facility, and Season also significantly interacted with an *R*^2^ value of 0.47 (Table S9), suggesting multiple factors are needed to capture trends in the fungal ecology of the built environment. The “Production Area” factor was especially informative, since it encompassed several other environmental conditions, including room temperature and humidity. For example, fungal swab sites within most Object Types, except Fans/Vents, clustered strongly by Production Area (Fig. 7A). *Post hoc* comparisons confirmed that fungal composition from Floor Objects, Doors, and Walls/Ceilings could significantly differ by Production Area (Table S10). Furthermore, YM counts could be associated with Object Type and Production Area (Fig. 7B), which can aid in predicting potential fungal reservoirs in the dairy processing environment. Future research on fungal presence and dissemination from fans and ventilation units in dairy processing environments is warranted. Fans/Vents harbored rich fungal diversity, with the single highest fungal richness value coming from a fan site (176, sample 17) (Table S3). In addition to being rarely cleaned, Fans/Vents circulate air that likely traveled throughout the processing environment, resulting in their diverse collection of surface fungi. Fungal dispersion from fans and ventilation units could circumvent the facility’s hygienic measures and contaminate product when doors between processing areas are opened, or there are changes in the facility’s air pressure, temperature, and humidity (50, 51). Furthermore, as the side-by-side swabbing results revealed, there was considerable microbial variability on individual fans themselves, suggesting that multiple sites on fans and ventilation units should be swabbed to identify fungal reservoirs.

While the fungal ecology of the built environment was somewhat specific to each dairy processing environment, our results demonstrate that Fans/Vents, Walls/Ceilings, Floor Objects, and Doors can harbor fungal reservoirs. To better understand the origins of spoilage fungi in the built environment, future studies should work to (i) refine metadata collection for environmental surface swabs, including additional object-specific parameters (e.g., sub-part and soil on surface), (ii) better differentiate seasonal from spatial variation in fungal ecology metrics through repeated time-series analysis, and (iii) pursue species- and strain-level identification of fungi in both products and the built environment. These recommendations can help develop methods to predict ecological outcomes in the dairy processing built environment from temporal, spatial, or physical parameters (52, 53). The outcomes from our study establish how environmental conditions, including Season, Production Area, and Object Type, should be taken into consideration for management measures to identify and control spoilage fungi in dairy processing environments.

### Conclusion

Our study demonstrates that Fans/Vents, Walls/Ceilings, Doors, and Floor Objects in cultured dairy facilities are conducive to fungal harborage. Indeed, many genera identified in the built environment were also present in spoiled product, including *Aureobasidium*, *Candida, Penicillium, Mucor,* and *Yarrowia*. These genera were also overrepresented at one specific swab site within facilities, suggesting that certain locations can act as point sources for product contamination. Surfaces from Fans/Vents demonstrated high fungal richness and warrant further investigation of their ability to disseminate fungi into facility air, which could circumvent hygienic zoning measures. Fungal diversity and genera were also significantly different by season, which can further inform fungal reservoir prediction. These findings can guide future environmental monitoring strategies to locate and control fungal reservoirs in dairy processing environments.

## MATERIALS AND METHODS

### Sample collection and processing

#### Environmental surface sample collection and processing

We collected environmental surface swabs and cultured dairy products from three facilities in New York State from July 2022 to June 2023. Facility 1 (178,000 ft^2^) produced a variety of cheese, sour cream, and yogurt products. Facilities 2 (42,000 ft^2^) and 3 (121,477 ft^2^) both produced sour cream and yogurt products. Facility 3 was the oldest building, with initial portions constructed nearly 100 years prior to the construction of Facilities 1 and 2. During the 2022–2023 sampling period, Facilities 1, 2, and 3 had approximately 195, 40, and 100 personnel employed, respectively.

**Fig 8:**
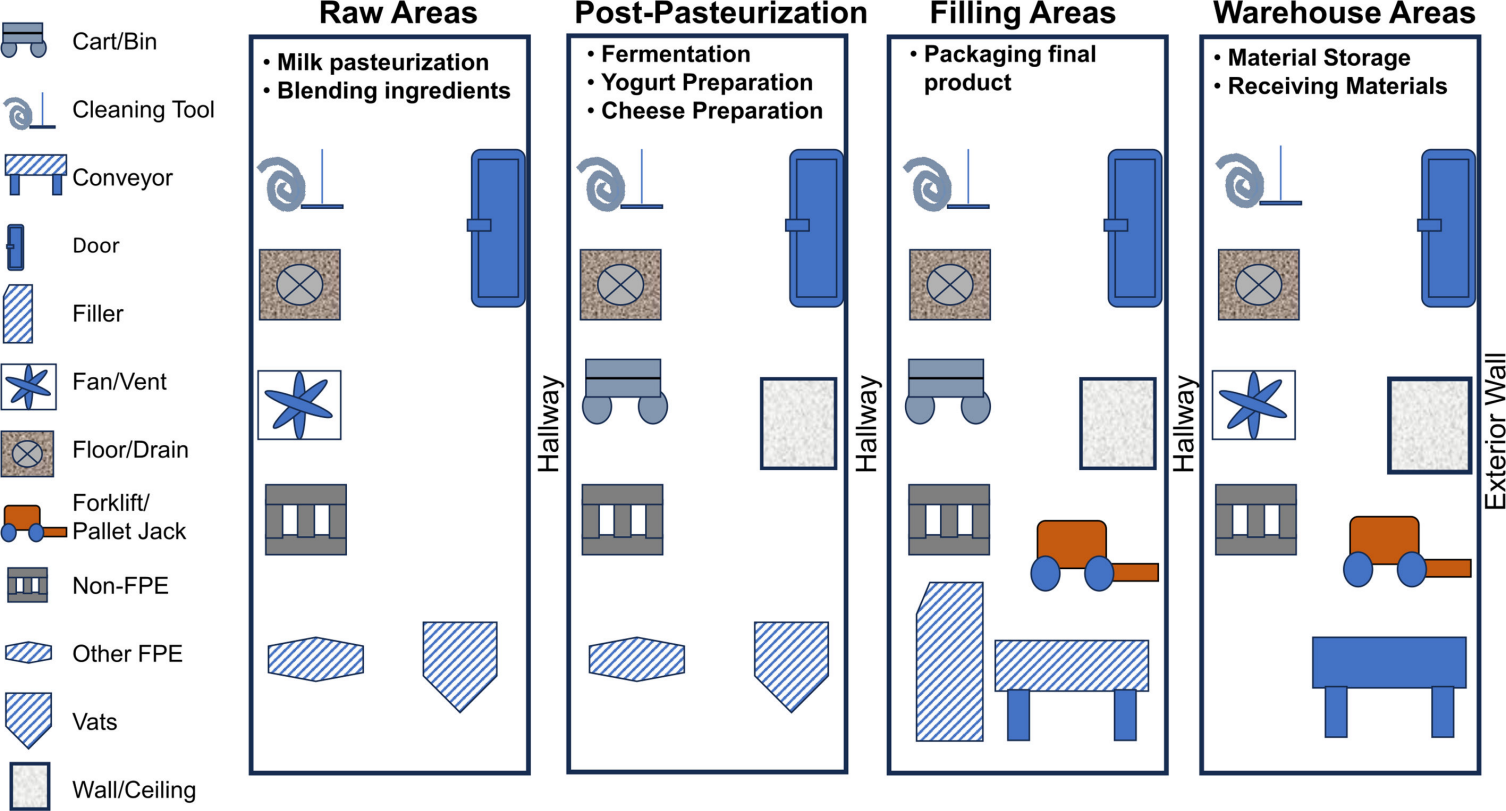
Description of areas and objects swabbed across three facilities. Diagonal lines indicate a Zone 2 object. Solid fill indicates Zone 3 objects. FPE, food processing equipment.

We collected 119 surface swabs from four Object Types, which were Doors (*n* = 35), Fans/Vents (*n* = 16), Floors (*n* = 48), and Walls/Ceilings (*n* = 20) (Fig. 8; Table S8). These swab sites were located in Warehouse (*n* = 65), Post-Pasteurization (*n* = 27), Filling (*n* = 12), and Raw areas (*n* = 15) (Fig. 8; Table S8). Our study examined Fans/Vents, Floors, Walls/Ceilings, and Doors because they were architectural structures of the building that did not contain food contact surfaces. Surfaces selected for swabbing on these structures were considered Zone 3 sites (Table S3). Each facility was sampled a total of four times (i.e., Summer [*n* = 39], Fall [*n* = 30], Winter [*n* = 21], and Spring [*n* = 29]). In subsequent sampling trips at each facility, the same or similar surfaces were swabbed as in the previous visits.

To sample environmental surfaces, we swabbed 10 vertical and 10 horizontal strokes over an approximate 1 square foot area using sponges (Neogen, Lansing, MI, USA) pre-moistened with Dey/Engley buffer (54). Swabs were promptly sealed in Whirl-Pak (Pleasant Prairie, WI, USA) bags, stored in coolers, and processed within 24 h. Samples were diluted and homogenized with phosphate-buffered saline (PBS). For YM enumeration, we prepared serial dilutions and cultured them on malt extract agar (MEA) plates, incubating at 25°C for 72 h. Colonies were expressed as log_10_ CFU/sponge.

#### Spoiled product collection and processing

Between October 2022 and June 2023, we received 28 shipments comprising 166 individual cultured dairy products from each facility. These products were in their original containers and had been sealed with clear tape if they had been opened. Products from each facility had been flagged for analysis due to visual defects, such as container bloating or visible YM growth, which had been identified through stress testing, shelf-life failure, or customer returns. Overall, 22 products were received from Facility 1, 15 from Facility 2, and 129 from Facility 3. Upon receiving these cultured dairy products, we stored them at 4°C and processed them within 24 h.

Visible mold growth on products (*n* = 154) was aseptically transferred to sterile Whirl-Pak bags (Whirl-Pak Nasco, Fort Atkinson, WI, USA). Products with no visible mold growth were homogenized. Then, 25 g from each product was transferred to a Whirl-Pak bag and diluted 10-fold with PBS. Each bag was homogenized with a Stomacher 400 Circulator Lab Blender (Stomacher 400 Circulator Lab Blender, Seward Technology Centre, West Sussex, UK) for 2 min at 230 RPM. For each bag, six 10-fold serial dilutions were then plated on MEA and incubated at 25°C. Isolates with distinct morphologies were selected and cultured on MEA plates. All pure cultures were stored in 20% glycerol solution at –80°C.

### Metadata collection

During environmental surface swabbing, we recorded metadata characteristics from each environmental site according to reference 54 (Table S3). Metadata included information about season, facility (age, personnel, year constructed, and building use history), production area (sanitation practices and humidity), and object (type, zone, and surface moisture). The surface was determined to be “dry” if there was no visible liquid on the surface, and “wet” if there was water or condensation on the surface. The relative humidity and temperature were measured for each room in the facility using a portable humidity meter (Omega, CT, USA). Humidity was categorized into the following ranges: “Very Humid” (>80%), “Humid” (>50%–80%), “Standard” (30%–50%), and “Standard Dry” (<30%).

For spoiled product collection, we recorded the type of product (i.e*.*, fruit yogurts, cottage cheese, and sour cream), the facility it was sourced from, and visible product defects according to reference 55 (Table S5).

### DNA extraction and ITS sequencing

For surface swabs, DNA extraction was performed using the Qiagen DNeasy PowerSoil DNA extraction kit (Qiagen, Germantown, MD, USA) with slight modifications to the manufacturer’s protocol. The homogenized sample mixture was centrifuged at 6,500 × *g* at 4°C for 20 min. The pellet was transferred to the PowerBead tube with CD1 buffer for bead-beating. For spoiled products, incubated pure culture plates were flooded with 2 mL PBS. Then, fungal mycelium and spores were scraped off the agar and homogenized with a sterile L-shape spreader. The culture suspension was then transferred to a PowerBead tube.

After transfer to the PowerBead tube, samples underwent DNA extraction using the DNeasy PowerSoil Pro Kit. The DNA concentration was measured using the NanoDrop 2000c UV-Vis Spectrophotometer (Thermo Scientific, Wilmington, DE, USA). Samples were removed if they had DNA concentrations lower than the detection limit of Qubit (i.e., 50 ng/mL). The primers ITS5 (5′-GGA AGT AAA AGT CGT AAC AAG G-3′) and ITS4 (5′-TCC TCC GCT TAT TGA TAT GC-3′) were used to amplify both the ITS1 and ITS2 regions using Illumina (56, 57). The nuclear ribosomal internal transcribed spacer region has been used as a universal DNA barcode marker for fungi (56, 57). Amplicons were barcoded with unique index adaptors through PCR (Novogene, China).

### ITS sequence processing and analysis

#### Environmental surface samples

Raw fastq files were demultiplexed, quality filtered, and analyzed with QIIME 2 (22.2) (v. qiime-2022.2) (58). Paired-end reads were denoised, chimeras were removed, and truncated at sites with a Phred quality score below 20 with DADA2 (59; Tables S18 and S19). Operational taxonomic units (OTUs) were clustered at 99% similarity using the VSEARCH plugin (60) according to reference 61. A naive Bayes classifier, trained on the UNITE database (62), classified the OTU sequences. Genera with fewer than 10 OTUs were removed from the data set.

Data were transformed to compositional form before analysis. Alpha diversity was calculated using the Shannon and Chao1 indices using the plot_richness function in the microbiomeutilities package (63) in RStudio. The Chao1 index measures community richness (number of species). The Shannon index measures species richness and evenness. Beta diversity was calculated using the Bray-Curtis (64) index in the betadispr function in the vegan package (65) in RStudio.

The qiime2R package was employed to import and analyze QIIME2 artifacts in RStudio (66). Fungal relative abundances at the genus level were calculated using the phyloseq package (67). Data visualization was performed using ggplot2 (68), while diversity analyses were conducted with the vegan package (65) in RStudio. Data tidying was facilitated by the tidyverse suite of packages (69).

#### Spoiled product

For cultured dairy isolates, raw Sanger sequencing reads in ab1 format were imported to Geneious (70) for *de novo* assembly. The primers used for Sanger sequencing were ITS5 (5′-GGA AGT AAA AGT CGT AAC AAG G-3′) and ITS4 (5′-TCC TCC GCT TAT TGA TAT GC-3′) to amplify both the ITS1 and ITS2 regions using Illumina. Sequence ends were trimmed and aligned using the Geneious Alignment algorithm. Assembled consensus reads were compared with the National Center for Biotechnology Information database using BLAST. The genera with the highest total score were recorded as a putative identification (>95%).

### Statistical analysis

Co-occurrence network analysis using the Bray-Curtis index for correlation was used to determine similarity among swab sites within each Production Area using the igraph package (66) in RStudio. Networks of individual surface swabs and edges were connected if they had >20% similarity.

Welch’s ANOVA *t* tests were performed in RStudio on YM counts generated for individual environmental samples, and ANOVA *t* tests were performed in RStudio on diversity values (71) (Table S16).

Principal coordinate analysis (PCoA) was performed to evaluate the distribution patterns of fungal samples using beta-diversity calculated by Bray-Curtis distance determinations performed with the phyloseq package (67) in RStudio.

PERMANOVA and *post hoc* pairwise comparisons were conducted using distance matrices with 999 permutations to determine statistically significant differences by the use of the “adonis” function in the “vegan” package (65) in RStudio. Multivariate homogeneity of group dispersions was assessed using Bray-Curtis distance matrices, and PCoAs were plotted; the centroids and covariance of each plotted factor were calculated to determine the effect of category (i.e*.,* Object, Facility, Season, and Production Area) on microbial diversity of the built environment and objects within it. The *P* values in the pairwise comparisons were corrected using false discovery rate.

Each environmental surface sample was binned into one of three microbial density categories, “Low,” “Medium,” or “High” (Table S3) based on its YM count. “Low density” consisted of values equal to or below 3 log_10_ CFU/Sponge. “Medium density” consisted of YM between 3 and 5 log_10_ CFU/Sponge. “High density” consisted of YM ≥5 log_10_ CFU/Sponge.

Chi-squared analysis was performed using the chisq.test function in the stats package (72) to assess the correlation of residuals between Object Type and YM density categories. All analyses were conducted using R software, version 4.3.1 (73).

Differential abundance analysis was conducted using the microbiomeSeq package in RStudio with the DESeq implementation (74). Taxa were examined at the genus level, and a 0.05 *P* value was the cutoff threshold.

For all analyses, a *P* value below 0.05 was considered to be significant.

### Side-by-side swabbing

To test the variability of fungi on surfaces, we conducted a separate experiment where we swabbed eight additional sites from each Object Type from Facility 1 in Spring 2024. From each surface, we swabbed two adjacent locations (i.e*.,* left and right). The surface swabs were processed, and the sequences were analyzed in the same manner as the other environmental surface samples.

## Data Availability

The data that support the findings of this study are available in the supplemental material of this article. The sequencing data from this study are openly available at the BioProject accession number PRJNA1123599. The software code from this study is openly available in the GitHub repository at https://github.com/dalysarah/Reservoirs-of-spoilage-fungi-in-the-built-environment-of-cultured-dairy-facilities.

## SUPPLEMENTAL MATERIAL

Figures S1 and S2 spectrum.00413-26-s0001.docx Residuals of the Chi-square test.

Supplemental tables spectrum.00413-26-s0002.xlsx Table S1 to S18.

Table S19 spectrum.00413-26-s0003.xlsx Summary of sequencing read processing. For each sample, the total number of input reads is shown alongside reads retained after quality filtering, denoising, merging of paired-end reads, and chimera removal. Percentages represent the proportion of the original input reads retained at each respective step.

ASM does not own the copyrights to Supplemental Material that may be linked to, or accessed through, an article. The authors have granted ASM a non-exclusive, world-wide license to publish the Supplemental Material files. Please contact the corresponding author directly for reuse.
