# Supplementary material for: Reservoirs of spoilage fungi in the built environment of cultured dairy facilities vary by season and location
Source: Microbiol Spectr. 2026 Jul 17;14(8):e00413-26. doi: 10.1128/spectrum.00413-26 (PMC13436190; doi:10.1128/spectrum.00413-26)
Supplement: Supplementary file 1 [file spectrum.00413-26-s0001.docx]

**Figure S.1***:* Residuals of the Chi Square test between Facility 1, 2, and 3 all Zone 2 and Zone 3 swabbed surfaces and YM Counts, Surface Moisture, and Humidity. Humid/Very Humid is >60% humidity; Standard 30-60%, Standard Dry, <30%. Blue circles represent a positive association and red circles represent a negative association. The diameter and color of the circle corresponds to the strength of the association. The larger and darker the circle, the stronger the association .

**Figure S.2.** Residuals of the Chi Square test between Object and YM Counts, Surface Moisture, Humidity. Blue circles represent a positive association and red circles represent a negative association. The diameter and color of the circle corresponds to the strength of the association. The larger and darker the circle, the stronger the association association. “High” is defined as YM ≥ 5 log_10_ CFU/sponge; Medium is defined as 3-5 log_10_ CFU/sponge; Low is ≤ 3 log_10_ CFU/surface.
